# Stromal ING1 expression induces a secretory phenotype and correlates with breast cancer patient survival

**DOI:** 10.1186/s12943-015-0434-x

**Published:** 2015-08-27

**Authors:** Satbir Thakur, Arash Nabbi, Alexander Klimowicz, Karl Riabowol

**Affiliations:** Department of Biochemistry and Molecular Biology, University of Calgary, 3330 Hospital Drive, Calgary, T2N 4N1 AB Canada; Department of Medical Science, University of Calgary, 3330 Hospital Drive, Calgary, T2N 4N1 AB Canada; Functional Tissue Imaging Unit, Translational Research Laboratory, Tom Baker Cancer Center, 1331- 29 street NW, Calgary, T2N 4N2 AB Canada; Present Address: Boehringer Ingelheim Pharmaceuticals Inc, 900 Ridgebury Road, Ridgefield, CT 06877 USA; Department of Oncology, University of Calgary, 311 HMRB, 3330 Hospital Drive, NW, Calgary, T2N 4N1 AB Canada

**Keywords:** Luminal breast cancer, Tumor suppressor, Biomarker, Stroma, AQUA, MMP/TIMP

## Abstract

**Background:**

Previous studies have established that levels of the Inhibitor of Growth 1(ING1) tumor suppressor are reduced in a significant proportion of different cancer types. Here we analyzed levels of ING1 in breast cancer patients to determine its prognostic significance as a biomarker for breast cancer prognosis.

**Methods:**

We used automated quantitative analysis (AQUA) to determine the levels of ING1 in the tumor associated stromal cells of 462 breast cancer samples. To better understand how high ING1 levels affect nearby epithelium, we measured the levels of cytokines and secreted matrix metalloproteases (MMPs), using an ELISA based assay in mammary fibroblasts overexpressing ING1. These cells were also used in a 3-dimensional co-culture with MCF7 cells to determine the effect of released MMPs and other cytokines on growing colonies.

**Results:**

We find that high levels of ING1 in stroma are associated with tumor grade (*p* = 0.001) and size (*p* = 0.02), and inversely associated with patient survival (*p* = 0.0001) in luminal, but not in non-luminal cancers, suggesting that high stromal ING1 promotes cancer development. In this group of patients ING1 could also predict patient survival and act as a biomarker (HR = 2.125). While ING1 increased or decreased the expression of different cytokines, ING1 also increased the levels of MMP1, MMP3 and MMP10 by 5–8 fold, and concomitantly decreased levels of the tissue inhibitors of metalloproteases TIMP2, TIMP3 and TIMP4 by 1.5–3.3 fold, resulting in significant increases in MMP activity as determined by zymography. Co-culturing of MCF7 cells with stromal cells expressing ING1 in 3-dimensional organoid cultures suggested that MCF7 colonies were less well defined, suggesting that secreted MMPs might promote migration.

**Conclusion:**

These data indicate that stromal ING1 expression can predict the survival of patients with luminal breast cancer. High levels of ING1 in stromal cells can promote the development of breast cancer through increased expression and release of MMPs and down regulation of TIMPs, which may be an underlying mechanism of reduced patient survival.

**Electronic supplementary material:**

The online version of this article (doi:10.1186/s12943-015-0434-x) contains supplementary material, which is available to authorized users.

## Background

Progression of tumors towards a malignant phenotype is not exclusively dependent on the autonomous properties of cancer cells, but is also influenced by the surrounding stroma. Tumor microenvironment which includes the extracellular matrix, surrounding blood vessels, endothelial cells, cancer-associated fibroblasts (CAFs), macrophages and other inflammatory cells, plays an important role in cancer progression. As a cancer progresses, its surrounding microenvironment co-evolves with it and attains an activated state through continuous paracrine communication, which creates a dynamic signaling interaction that promotes cancer initiation and growth [1].

The most prominent cell types in the tumor stroma are the cancer-associated fibroblasts. CAFs are heterogeneous populations and their relative composition differs among different tumor types [2]. The mechanisms that can activate CAFs in the stroma are still not clearly understood but it is believed that tumor released factors such as tumor growth factor beta (TGF-β), platelet derived growth factor α/β (PDGFA, PDGFB), fibroblast growth factor (FGF) and interleukin-6 (IL-6) play major roles in trans-differentiation and activation of CAFs [3–6]. Activated CAFs are known to contribute to tumor progression by various cellular mechanisms. These cells produce and release several hormones and cytokines such as epidermal growth factors, fibroblast growth factors, IL-6 etc., that can stimulate cancer cells to proliferate rapidly [7]. While CAFs are well characterized for supporting tumor progression, a few studies have also reported their cancer-initiating capacity [8]. In addition to providing growth cues to cancer cells, CAFs also contribute in evading apoptosis by constantly providing them with survival signals like cytokines and insulin like growth factor (IGF) [9]. They are also known to produce various matrix components like collagen, which makes the extracellular membrane (ECM) more cross-linked and has been shown to enhance integrin signaling in cancer cells, which in turn can activate pro-survival PI3K/AKT pathways downstream [10]. CAF secreted cytokines and chemokines also lead to the infiltration of various pro-inflammatory immune cells, which can promote angiogenesis and metastasis [11]. Particularly, CAF released factors such as stromal derived factor-1 (SDF-1), vascular endothelial growth factor (VEGF), IL-8, IL-6, and IL-1β cooperate and promote new vessel formation by recruiting endothelial cells [12].

CAFs also secrete several members of the matrix metalloproteases (MMP) family. These enzymes can degrade ECM, which helps tumor cells invade the surrounding tissues. MMPs can also cleave membrane bound growth factors like VEGF and cytokines as well as their receptors and cell adhesion molecules like cadherins, which can lead to increased motility and result in epithelial to mesenchymal transition (EMT) [13, 14]. As stroma derived factors promote initiation, growth and progression of tumor cells, they can also determine the therapeutic outcome in patients as they can act as barriers to therapy [15].

Senescence can also affect tissue microenvironment reactivity as well as secreted factors from CAFs. Senescent fibroblasts acquire a senescence-associated secretory phenotype (SASP) which turns senescent fibroblasts into pro-inflammatory cells allowing them to increase their pro-inflammatory and pro-angiogenic cytokine and chemokine production. SASP induction in stromal fibroblasts has been positively correlated with tumor progression and it has been observed that they show this effect by inducing EMT in nearby epithelial cells [16]. Along with various cytokines, senescent cells also secrete increased levels of some MMPs. The MMP family members that are consistently upregulated in senescent fibroblasts are MMP-1, MMP-3 and MMP-10 [17]. These increased levels of secreted MMPs can degrade the components of the extracellular matrix, which can affect the physical property of the tissue structure. This could help tumor cells migrate and invade through the ECM. It has also been observed that senescent cells and malignant tumors share many common repertoires of MMPs indicating the significance of senescence induction in tumor cell metastasis.

All ING family members (ING1-5) have been reported to be altered in localization, sequence, or expression level in various cancer types and are classified as type-II tumor suppressors [18]. The human ING1 gene encodes four different isoforms, p47ING1a, p33ING1b, p24ING1c, and p27ING1d, among which p47ING1a and p33ING1b are the best characterized so far [19, 20]. ING gene products possess distinct, but in some cases overlapping functional properties and unique expression profiles in eukaryotic systems [18, 21]. Ectopic overexpression of ING1 has been found to block cell cycle progression by arresting cells in G1 phase of the cell cycle, and long term expression promotes apoptosis. Particularly, overexpression of p47ING1a is known to induce senescence like characteristics including high SA-β-gal activity, presence of SAHF, altered nuclear morphology, increased expression of p16 and Rb and growth arrest [22], whereas, p33ING1b overexpression induces cells to undergo apoptosis [23]. Consistent with a role as a tumor suppressor, inhibition of ING1 expression with antisense RNA promotes focus formation *in vitro* and tumor formation *in vivo* [24, 25]. Loss of ING1 expression has been implicated in a broad range of human cancer types, including primary breast tumors, lymphoid malignancies, testis tumors, squamous cell cancers, and head and neck cancers [18, 26–28]. Other members of the ING family, and in particular ING4, have also been reported to be down-regulated in breast cancers with a dominant mutant allele of the *ING4* gene promoting tumor growth [29, 30]. Recently, we reported that reduced ING1 levels are correlated with increased metastasis in breast cancer patients [31].

Here we asked if ING1 expression could predict breast cancer patient outcome using an automated quantitative immune-histological technique to determine ING1 expression in the tumoral and stromal compartments of patient tissue samples. We found that stromal expression of ING1 showed an inverse correlation with patient survival. ING1 expression also correlated with tumor grade in these patients and multivariate analysis showed that ING1 was an independent prognostic marker in the breast cancer cohort we tested. Furthermore, we found that ING1 expression can regulate the levels of various cytokines, matrix metalloproteases and their inhibitors, tissue-inhibitors of matrix metalloproteases in mammary fibroblasts that could explain partly, at least, the inverse correlation between the stromal ING1 expression and patient survival. Overall, this study provides important pre-clinical data that could help establish ING1 as a prognostic and therapeutic agent for breast cancer.

## Results

### Stromal ING1 expression in breast cancer patient samples

**Fig. 1:**
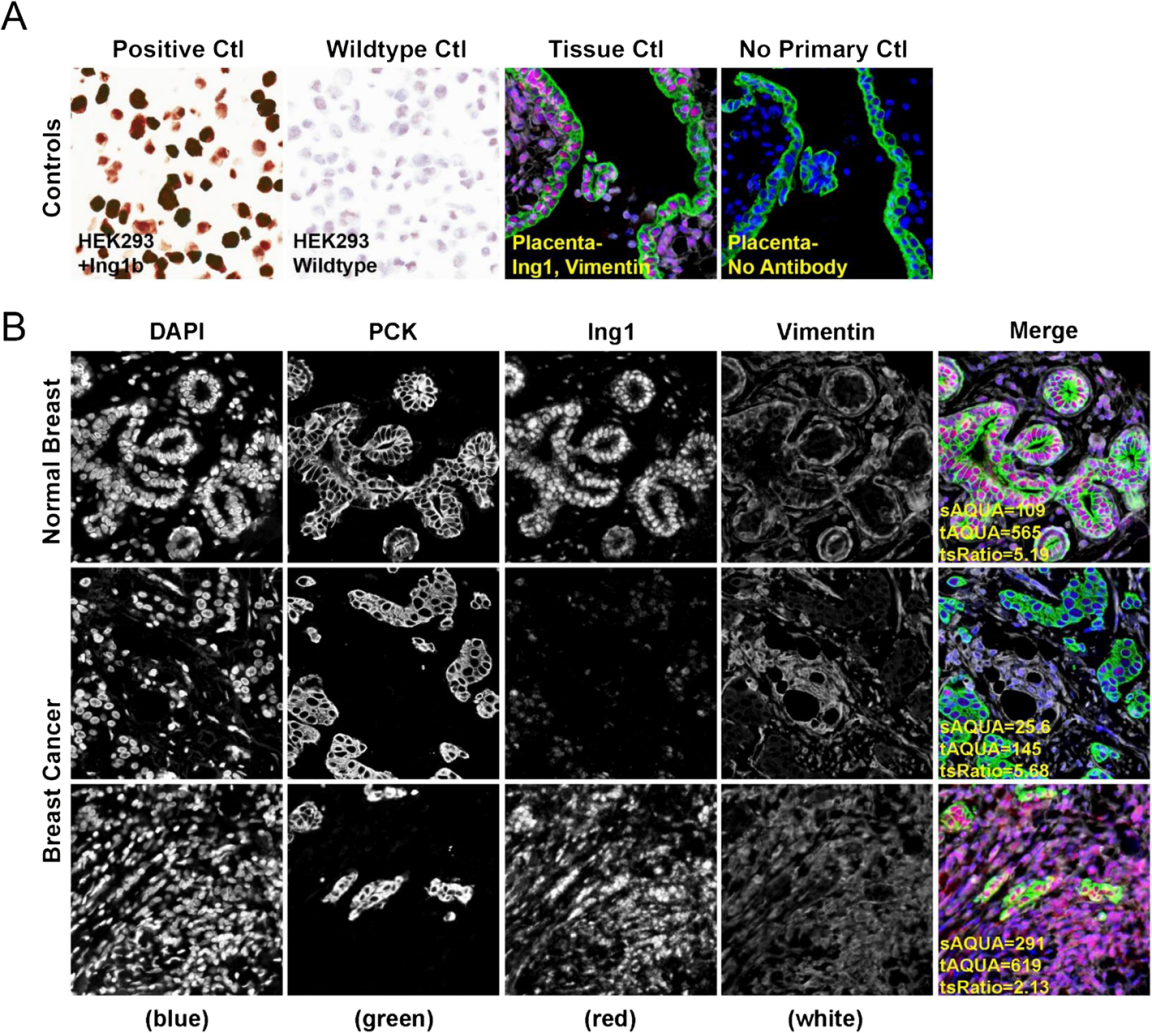
Immunohistochemical staining and quantitation of stromal ING1 using the HistoRx AQUA platform. **a** Representative images showing specificity of the ING1 monoclonal antibody in HEK293 cells and HEK293 cells overexpressing ING1a (*left* panels) and in placenta treated with or without the ING1 antibody (*right* panels). **b** Representative examples of quantitative fluorescent IHC images for ING1 expression in normal breast tissue (*top row* of panels) and breast cancer tissue (*two bottom rows* of panels). tAQUA scores represent the expression level of ING1 within the pan-cytokeratin defined epithelial/tumor compartment; sAQUA scores represent expression level of ING1 in the vimentin defined mesenchymal/stromal compartment. DAPI-stained nuclei are depicted in blue, pan-cytokeratin stained epithelial/tumor cells are depicted in green, ING1 protein expression is depicted in red and vimentin stained mesenchymal/stromal cells in white

ING1 protein level was measured using quantitative fluorescence immunohistochemistry on the HistoRx AQUA® platform in breast cancer patient samples from the Calgary Tamoxifen cohort as described previously [31]. The specificity of the ING1 monoclonal antibody used for fluorescence IHC was assessed in HEK293 cells and placental tissue Fig. 1a (top panel). The patient samples were also stained with anti-pan cytokeratin and anti-vimentin antibodies to specifically demarcate the tumor region from the stromal region respectively. As our focus was on the expression of ING1 protein in the stromal region of breast cancer patients, we used the expression of ING1 in the vimentin positive region of normal breast tissue sample as our baseline control (Fig. 1b top panel). The ING1 localization was found to be primarily nuclear in these regions with a mean AQUA score of 109, which was used as a cut point to dichotomize patients. In breast cancer patient samples, varying levels of ING1 expression were found in the stromal (vimentin positive) regions, which were quantified and then used for classifying patients with low stromal or high stromal ING1 expressing tumors.

Figure 1b middle panel shows a representative image of a sample with low stromal ING1 expression (AQUA score 25.6) and the bottom panel shows representative images of a patient sample with high stromal ING1 expression (AQUA score 291).

### Prognostic value of stromal ING1 expression in breast cancer patients

As described previously, the cohort tested in this study has patients classified into luminal breast cancer (ER positive and Her2 negative, *n* = 430) and non-luminal breast cancer (ER negative or Her2 positive, *n* = 32) groups for analysis. We tested the prognostic value of stromal ING1 expression in both populations.

**Fig. 2:**
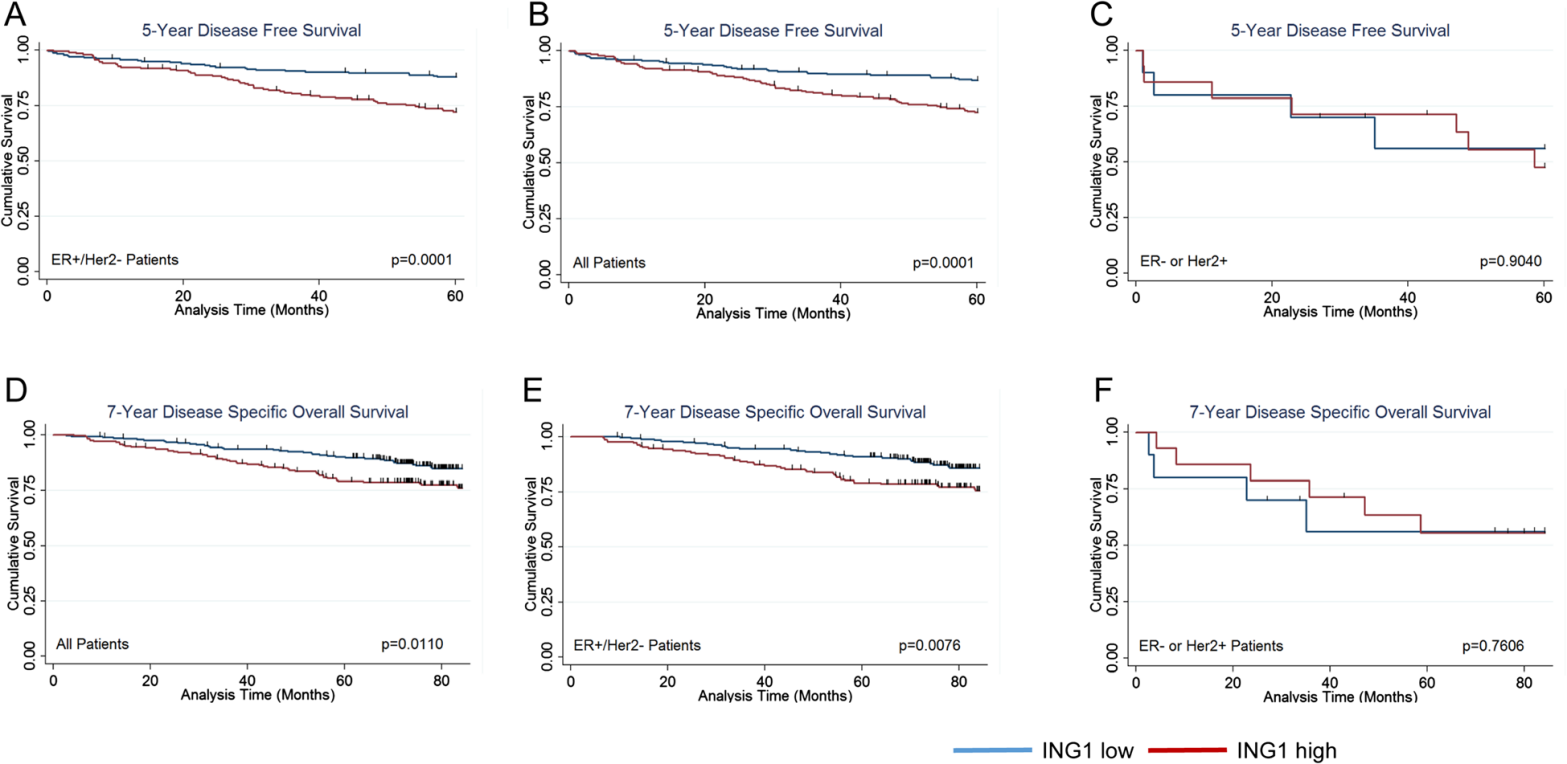
Kaplan-Meier survival analysis. **a**-**c** Kaplan-Meier survival curves for Disease free survival (**a**) in total population (**b**) ER+/HER2- group (**c**) ER- or HER2+ group. **d**-**f** Kaplan-Meier survival curves for Disease specific overall survival (**d**) in total population (**e**) ER+/HER2- group (**f**) ER- or HER2+ group

Contrary to the observations made with ING1 expression in the tumor compartment [31], significant results were obtained in the luminal group. In this group, stromal ING1 expression correlated with clinico-pathological characteristics like tumor grade (*p* = 0.001) and tumor size (*p* = 0.020) whereas the non-luminal group did not show correlation to any of the clinic-pathological characteristics listed in Table 1. High stromal ING1 expression in the luminal group also correlated to poor survival outcomes in patients as indicated by Kaplan Meier analysis. This observation was made in both survival outcomes, including disease free survival (Fig. 2b, *p* = 0.0001) and disease specific overall survival (Fig. 2e, *p* = 0.0076). Interestingly, no differences in survival outcomes were seen in this analysis in the non-luminal group dichotomized by ING1 expression (Fig. 2c and f) which previously has shown that higher ING1 expression in the tumor compartment could predict better survival outcome for breast cancer patients [31]. This observation suggests that ING1 expression level in tumor and stromal regions could specifically predict survival of patients having different types of breast cancers.

**Table 1:** Association of clinico-pathological characteristics of ER+/HER2- breast cancer patients with levels of ING1 in the stroma

Characteristics	# of cases (%)	ER+/Her2-		
		Low lng1	High lng1	P-value
Age				
< 53	72 (16.25)	56	16	0.880
≥ 53	371 (83.75)	284	87	
Menopausal status				
Pre-menopausal	26 (5.90)	17	9	
Peri-menopausal	21 (4.70)	18	3	0.231
Post-menopausal	321 (72.50)	242	79	
n/a (male)	1 (0.20)	1	0	
Unknown	74 (16.70)	62	12	
Stage				
I	188 (42.40)	148	40	0.365
II	138 (31.20)	105	33	
III	36 (8.20)	24	12	
IV	5 (1.10)	3	2	
Unknown	76 (17.20)	60	16	
Tumor grade				
1	103 (25.40)	88	15	0.001
2	247 (60.80)	195	52	
3	56 (13.80)	31	25	
Tumor size				
< 2 cm	223 (53.86)	179	44	0.020
≥ 2 cm	191 (46.14)	137	54	
Lymph node status				
Negative	278 (72.77)	220	58	0.103
Positive	104 (27.23)	74	30	
Rx Tamoxifen				
No	161 (37.44)	118	43	0.196
Yes	269 (62.56)	212	57	

The listed clinico-pathological characteristics were analyzed for their correlation with low/high levels of stromal ING1. Stromal ING1 shows a correlation with tumor grade and size in the ER+/ Her2- group of patients in the cohort

Next, a Cox proportional hazards model was used to assess the independent prognostic value of stromal ING1 in the cohort. This analysis was performed to determine if any of the clinically relevant biomarkers along with stromal ING1 levels has a strong prognostic/predictive ability regarding disease free survival in the cohort. Established biomarkers such as tumor grade, tumor size, lymph node status, ER levels and HER2 status were included in the multivariate model along with stromal ING1 levels, since these variables are routinely used clinically. The variables included in the analysis were compared for their hazard ratio (HR), which indicates the prognostic power of a given biomarker. In the analysis, tumor grade [HR 2.741, *p* = 0.002], lymph node status [HR 3.505, *p* < 0.001] and stromal ING1 [HR 2.320, *p* = 0.002], were significantly and independently associated with disease free survival in ER+/HER2- population (Table 2). This suggests that stromal ING1 levels are equal in predictive power to the established variables of tumor grade and lymph node status in the cohort tested in this study.

**Table 2:** Multivariate analysis of disease free survival

Variable	Hazard ratio	*P* value	9 % CI
All patients			
Tumor grade	2.741	< 0.001	1.618–4.647
Tumor size	1.431	0.188	0.840–2.437
Lymph node status	3.505	< 0.001	2.142–5.737
ER	0.360	0.010	0.165–0.785
HER2	1.669	0.391	0.518–5.383
Stromal 1NG1	2.125	0.004	1.270–3.557
ER+/HER2-patients			
Tumor grade	2.447	0.002	1.374–4.358
Tumor size	1.401	0.236	0.802–2.446
Lymph node status	3.429	<0.001	2.014–5.839
Stromal 1NG1	2.320	0.002	1.356–3.969

Multivariate analysis of clinically relevant biomarkers along with stromal ING1. Stromal ING1 level is a significant and independent biomarker in the total population and ER+/HER2- sub-population in the Calgary Tamoxifen Cohort

### ING1 regulates levels of cytokines produced in mammary fibroblasts

**Fig. 3:**
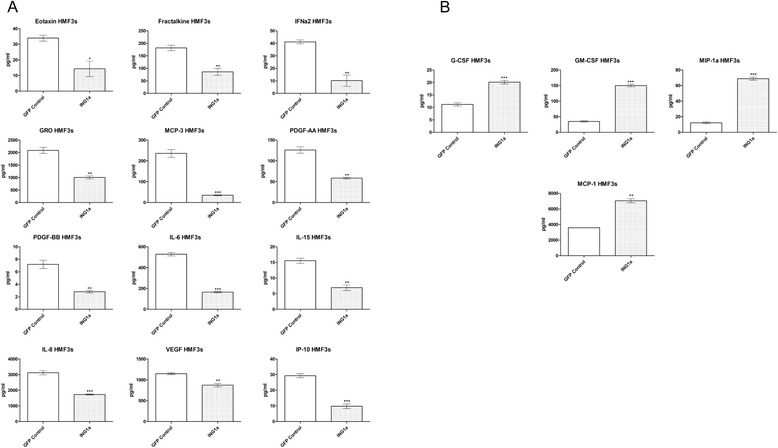
Cytokine profile of HMF3s upon ING1a overexpression. **a** Cytokines showing decrease in concentration upon ING1a overexpression in HMF3s cells as compared to GFP control (*n* = 3; * p < 0.05; ** *p* < 0.001; *** *p* < 0.0001). **b** Cytokines showing increase in concentration upon ING1a overexpression in HMF3s cells as compared to GFP control (*n* = 3; ** *p* < 0.001; *** *p* < 0.0001)

In the stroma, fibroblasts are the most abundant cell type and are the most active cellular component of cancer-associated stroma [32]. They are believed to play active roles in the promotion of processes like angiogenesis, metastasis and overall tumor growth through expressing various paracrine factors [7, 11, 12]. As high stromal ING1 expression significantly correlated with poor patient survival in the breast cancer cohort tested in this study, we next determined what cytokines might be regulated by ING1 in the stroma. For this, ING1a was overexpressed using adenoviral vectors in the human mammary fibroblast cells (HMF3s) and the conditioned media from these cells was collected and analyzed for various cytokines/chemokines using an ELISA based assay. ING1a was chosen, since it is believed that senescing stromal cells contribute to the induction of cancers *in vivo* and we here found that of the ING1 isoforms, ING1a is most effective in inducing cellular senescence [22]. Figure 3a shows several cytokines that showed a significant decrease in levels upon ING1a overexpression compared to GFP overexpressing HMF3s cells. In contrast, some cytokines in the panel were upregulated upon ING1a overexpression (Fig. 3b), the mechanism of which needs to be further investigated.

**Fig. 4:**
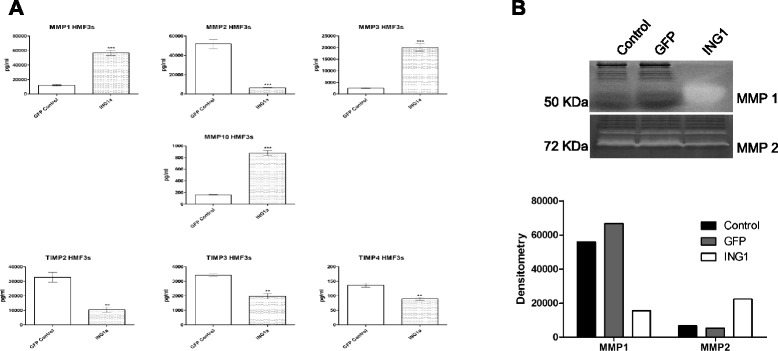
MMPs/TIMPs regulated by ING1a in HMF3s cells. **a** Levels of MMPs and TIMPs upon ING1a overexpression in HMF3s cells (n = 3; ** *p* < 0.001; *** *p* < 0.0001). **b** Zymographs depicting caseinolytic and gelatinolytic activity of MMP-1 and MMP-2 respectively in HMF3s cells. The *bottom* panel shows densitometric analysis of zymographs

Tumor associated stroma is also known to produce a plethora of matrix metalloproteases (MMPs), which act on the cell surface of cells and help tumor cells to invade surrounding tissue and metastasize to distant regions to form secondary tumors. Taking this into consideration, we tested for changes in the levels of various MMPs and their inhibitors, tissue inhibitors of metalloproteases (TIMPs). There were significant changes in the amount of all MMPs examined in HMF3s cells overexpressing ING1a, with simultaneous decrease in the levels of inhibitory TIMPs. Figure 4a shows the MMPs and TIMPs, which show a significant change in levels with respect to ING1 overexpression in HMF3s cells. With the exception of MMP-2, all other MMPs increased in levels while TIMPs decreased in cells overexpressing ING1a, consistent with ING1 playing an active role in invasion and metastasis.

### Functional assay for MMPs regulated by ING1a in HMF3s cells

Since we saw a significant change in the levels of MMPs released by HMF3s cells upon ING1a overexpression, our next question was to determine the functional activity of the MMPs produced. The activation of MMPs is believed to be a key feature in inducing tumor invasiveness and metastasis both *in vitro* and *in vivo* and both *MMP-1* and *MMP-2* have been identified as genes associated with the ability of human breast cancer cells to metastasize spontaneously to the lungs in immune deficient mice [33]. In another study involving a mammary fat pad rat xenograft model, expression of MMP-1 in stromal fibroblasts was shown to confer high invasion potential to breast carcinoma cells [34].

Using zymography analysis, we determined the caseinolytic and gelatinolytic activity of MMP-1 and MMP-2 respectively in cells expressing ING1a. Results obtained from this experiment were in line with the results from ELISA based analysis as conditioned media from HMF3s cells overexpressing ING1a had greater MMP-1 activity compared to media from untreated cells and GFP only expressing cells (Fig. 4b). Similar to our previous observations, the activity of pro-MMP-2 was reduced in the conditioned media from ING1a overexpressing HMF3s cells in comparison to control and GFP expressing cells.

### ING1a overexpressing HMF3s cells induce disorganization of breast cancer cell derived organoids

In a physiological setting, cancer associated fibroblasts in the stroma are believed to release factors that help tumor cells to invade surrounding tissue, metastasize or divide more rapidly. In order to recapitulate this physiological phenomenon *in vitro* we employed a three dimensional culture system in which cells from an ER+ breast cancer cell line MCF7, and HMF3s fibroblast cells expressing ING1a, were grown separately in the context of matrix support to provide an environment that more closely reflects an *in vivo* setting. We then co-cultured MCF7 and HMF3s expressing ING1a in order to test whether paracrine factors released by HMF3s upon ING1a overexpression could affect organoids formed by MCF7.

**Fig. 5:**
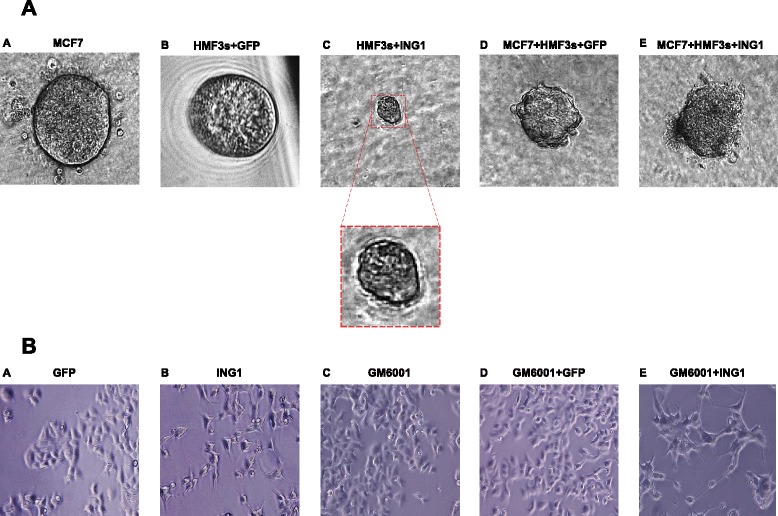
Three dimensional co-culture of MCF7 and HMF3s cells. Panel **a** ING1a induces disorganization of MCF7 breast cancer cell-derived organoids. MCF7 or HMF3s cells were grown individually or co-cultured in Matrigel in three-dimensional cultures in ultralow attachment 96-well plates. Representative images of day 14 (*a*) MCF7 cells alone (*b*) HMF3s cells expressing GFP (*c*) HMF3s cells expressing ING1a; inset magnified spheroid (*d*) MCF7 cells co-cultured with GFP expressing HMF3s cells (*e*) MCF7 cells co-cultured with HMF3s cells expressing ING1a captured by digital camera phase contrast microscopy. Panel **b** MMP blockade partially reverses ING1a induced morphology changes in MCF7 cells. MCF7 cells were supplemented with conditioned media from HMF3s cells infected with Ad-ING1a or Ad-GFP, alone or in combination with GM6001. Representative images after 24 h were captured using a phase contrast microscope

The organoids derived from both MCF7 and HMF3s cells were monitored at distinct time intervals by phase contrast microscopy. Non-transformed mammary epithelial cells are known to form spheres with lumens in three-dimensional cultures, that mimic mammary gland tissue [35]. We found that both MCF7 as well as control HMF3s cells expressing GFP formed filled spheres (Fig. 5a and b) in culture. This observation is expected with MCF7 being a breast cancer cell line derived from a metastatic site and the HMF3s fibroblast cell line being of mesenchymal origin. HMF3s cells overexpressing ING1a cultured alone also formed filled spheroids which were considerably smaller in size than those formed by MCF7 and GFP expressing HMF3s cells when cultured alone (Fig. 5*c*).

Three dimensional culture assays allow phenotypic discrimination between nonmalignant and malignant mammary cells. Nonmalignant cells grown in a three dimensional context form growth arrested acinus-like colonies, whereas malignant cells form disorganized colonies that continue to proliferate [36]. Similar results were obtained when HMF3s cells overexpressing ING1a were co-cultured with MCF7 cells (Fig. 5*e*). The colonies formed in this combination were more disorganized and distorted as compared to individual controls and when MCF7 cells were co-cultured with GFP-expressing HMF3s cells (Fig. 5a, panel *d*, Additional file 1: Figure S1).

To test if the aggressive colonies formed were due to higher amounts of secreted MMPs upon ING1a overexpression in HMF3s cells, we next examined the morphology of MCF7 cells supplemented with conditioned media from HMF3s treated with the broad specificity MMP inhibitor GM6001. MCF7 cells supplemented with conditioned media from ING1a expressing HMF3s cells show a clear change in morphology (Fig. 5b panel *b*) in line with our previous observations. This phenotype was largely reversed when cells were supplemented with media from ING1a expressing HMF3s cells treated with GM6001 (Fig. 5b panel *e*). Cells looked more fusiform compared to cells treated with ING1a conditioned media in the absence of the inhibitor.

Taken together, these data suggest that ING1a expression in mesenchymal fibroblasts induces release of certain paracrine factors, which further induce epithelial breast cancer cells to attain a more aggressive phenotype.

## Discussion

In this study we investigated the significance of ING1 expression in the stromal region of breast cancer patients and tested the prognostic/predictive value of stromal ING1 as a prognostic factor in the Calgary Tamoxifen Cohort. Prognostic markers are associated with outcomes independent from therapy whereas, predictive markers predict outcome in terms of survival of a specific therapy. Considering the fact that the patients in the cohort studied were treated with tamoxifen irrespective of the ER status, ING1 acting as a predictive or prognostic marker remains unclear. Results show that stromal ING1 correlates with clinico-pathological characters like tumor grade (*p* = 0.001) and tumor size (*p* = 0.020) in the luminal (ER+/HER2-) breast cancer group consisting of 443 patients in the cohort. Specifically, low stromal ING1 expression was associated with tumor grade and size, i.e. patients with lower expression of stromal ING1 had better prognosis. This is an interesting observation and in contrast to what was observed in the case of tumor ING1 expression. Therefore, it appears that high ING1 levels in tumor and low ING1 levels in stroma predict the best outcomes for patients.

We also investigated if stromal ING1 expression was associated with disease free survival and disease specific overall survival by analyzing survival outcomes using Kaplan Meier curves. Significant association with 5 year DFS were observed in both the luminal group (*p* = 0.0001) and in the total population (*p* = 0.0001) of the cohort, with patients expressing low stromal ING1 having a better prognosis than patients with high stromal ING1. Similar results were obtained when the association with 7 year DSOS was analyzed, with both the luminal group (*p* = 0.0076) and total population (*p* = 0.110) showing better prognosis than the low stromal ING1 category, although the relationship was less statistically significant. Patients from the non-luminal category were also analyzed for similar associations, but no significant results were obtained for DFS or DSOS, suggesting that ING1 expression in different compartments of tissue (tumor/stroma) may have different roles to play in promoting or inhibiting the development of different sub-types of breast cancers.

Furthermore, multivariate analysis using Cox proportional hazards regression to adjust for important clinical covariates, confirmed that stromal ING1 expression was independently associated with DSS in breast cancer. When taking the total population of the cohort into account, stromal ING1 (HR 2.125, *p* = 0.004) not only came out to be an independent prognostic marker for breast cancer, but had better predictive power than already established biomarkers like HER2, ER and tumor size, which are commonly used in the clinic. When the luminal only group was analyzed, similar results were obtained, where stromal ING1 (HR 2.32, *p* = 0.002) came out as an independent prognostic marker in the cohort tested. In this particular population, stromal ING1 had better predictive value than tumor size, which is a clinically used biomarker. These results indicate that stromal ING1 is not only associated with patient survival and clinico-pathological characters like tumor grade and size in breast cancer, but could also be developed into a biomarker for breast cancer considering its predictive value than currently used clinical biomarkers. Further testing needs to be done in this regard by validating the present observation in a different breast cancer cohort, along with clinical testing, to establish stromal ING1 as a bona fide biomarker in breast cancer.

As this observation of association of stromal ING1 with patient survival was unanticipated for a typical type-II tumor suppressor, we wanted to determine the underlying reason for association of higher stromal ING1 expression with poor prognosis of patients in the luminal group of the cohort. Tumor cells in a patient are surrounded by a complex microenvironment that includes the extracellular matrix, diffusible growth factors and cytokines, and a variety of non-epithelial cells like endothelial cells, pericytes, smooth muscle cells, immune cells and fibroblasts collectively called as stroma. The concept of growth regulatory interactions between the stroma and adjacent epithelial cell population was first introduced by Schor et al. [37, 38]. This interaction is mediated by soluble autocrine/paracrine factors secreted from stromal cells that induce physiological changes such as increased proliferation, migration etc. in the adjacent epithelia. Specifically, fibroblasts present in the stroma are known to produce several families of growth factors that are key mediators of stroma-tumor cell interactions. Since fibroblasts constitute the majority of the stromal cells within a breast carcinoma, we used a human mammary fibroblast cell line to examine changes in cytokines and growth factors produced by these cells upon modulating levels of ING1. One previous study reported a role for the ING4 member of the ING family in regulating the secretory phenotype of primary fibroblasts [39], which is interesting in light of the fact that the different ING family members are stoichometric members of both histone deacetylase (HDAC, INGs 1&2) and histone acetyltransferase (HAT, INGs 3,4,5) complexes, and they appear to have overlapping function in regulating the secretome.

Since ING1b induced cell death in HMF3s cells, we used ING1a, which is known to induce senescence [22] in our experiments to determine the cytokine profile of these cells. Various studies have provided evidence that senescent human fibroblasts can promote the proliferation of pre-malignant and malignant epithelial cells in culture, and the formation of tumors in mice [32, 40, 41]. This is likely due to the fact that senescent fibroblasts show a senescence-associated secretory phenotype (SASP), which is similar to the paracrine growth factors and cytokines made in tumors that can contribute to cancer progression. Contrary to our expectations, our results showed that there was a significant decrease in the number of pro-inflammatory and proliferative cytokines such as IL-6, IL-8, PDGFA, PDGFB, VEGF and GRO in HMF3s cells upon ING1a overexpression. Chemotactic cytokines promoting growth and recruitment of immune cells (neutrophils, monocytes and macrophage) like Eotaxin, Fractalkine and MCP-3 were also released at lower amounts compared to controls. However, IL-15 and IP-10 which are known to have anti-apoptotic and anti-angiogenic properties [42, 43] also had lower levels upon ING1a expression in HMF3s cells, suggesting that these pathways may be activated by senescing fibroblasts.

Apart from the majority of cytokines and chemokines having reduced levels, some cytokines were increased upon ING1a expression. An increase in the levels of immune cell (granulocyte, monocyte and macrophage) promoting cytokines such as G-CSF, GM-CSF and MIP-1a was also observed along with increases in chemotactic and pro-inflammatory cytokine MCP-1.

In contrast to the ING1a effects on cytokines and chemokines, a much more directed effect was seen for matrix metalloproteases. Stromal cells secrete matrix metalloproteases such as MMP-1, 2, 3, 9 and 10, all of which can promote epithelial transformation [44] and are known to have pro-angiogenic and metastatic properties [45, 46]. Previous studies have shown that senescent cells secrete increased levels of MMPs and the MMP family members that are consistently upregulated in fibroblasts undergoing senescence are MMP-1, 3 and 10 [17]. Our previous study shows that ING1a induces senescence suggesting that these MMPs may contribute to epithelial cell transformation. Significant increases in the levels of MMPs implicated in senescence upon ING1a overexpression in HMF3s cells also indicates senescence induction in these cells. A reciprocal effect was observed in the case of inhibitors of MMPs (TIMPs) as the levels of TIMP-2, 3 and 4 showed a significant decrease in ING1a expressing cells indicating that both MMP production increased and inhibitor of MMP activity had decreased in a coordinated manner in these cells.

These results clearly showed an increased amount of MMPs being secreted by the HMF3s cells expressing ING1a. To test if the MMPs secreted were active, we performed zymography with specific substrates for MMP-1 and MMP-2 to analyze their enzymatic activity *in vitro*. Activities on the zymogram corresponding to levels of MMP-1 and 2 previously observed were obtained confirming that the MMPs secreted by ING1a expressing HMF3s cells were biologically active.

Next, in order to functionally investigate whether soluble factors produced by fibroblasts upon ING1a expression are able to induce changes in tumor cells, we co-cultured HMF3s cells and MCF7 cells and studied their behavior in three dimensional cultures. The organoids formed when HMF3s cells expressing ING1a were co-cultured with MCF7 cells were highly disorganized as compared to GFP expressing co-cultured cells. This is an indication that the MMPs and cytokines secreted by ING1a expressing cells were able to induce morphological changes in the cancer cells resulting in disorganized and more aggressive colonies. Partial reversal of morphology of MCF7 cells when supplemented with conditioned media from ING1a and MMP inhibitor treated cells in comparison to MCF7 cells treated with ING1a only media, further indicates that MMPs play a role in MCF7 cells attaining a more aggressive phenotype in response to altered levels of ING1. The ability of ING1a expressing fibroblasts to stimulate the invasion of tumor cells into the matrix indicated that secreted factors produced by these cells in response to ING1a expression may be sufficient to induce invasiveness in these cells. This may explain the phenomenon believed to occur in tumor-stroma interactions in cancer patients, where senescent cancer associated fibroblasts release soluble factors which provides an environment that helps the tumors to grow more aggressively and to metastasize to form secondary tumors.

Overall, these results from the cytokine and MMP profiling and the functional assays provide a possible explanation for the poor survival of breast cancer patients having elevated levels of ING1 in tumor associated stroma as observed in the AQUA analysis of patients having luminal types of breast cancer in the cohort we tested. Both major isoforms of ING1, ING1a and ING1b have been reported to induce senescence in cells upon overexpression [22, 47, 48] although ING1b also induces apoptosis at higher levels while ING1a appears to solely affect senescence. Therefore, we speculate that increased levels of ING1 in the stroma may induce senescence which promotes stromal cells developing a senescence associated secretory phenotype, releasing paracrine and cytokine growth factors and MMPs. This would result in more aggressive tumor formation in such patients leading to their documented poor survival.

## Conclusions

Presently, a high incidence of breast cancer is observed in women worldwide. This is most likely due to the availability of widespread screening programs used to detect breast cancer, which otherwise may never get diagnosed. There is an inverse relation between the cost of treatment and patient survival as the breast cancer progresses to higher grades which makes screening and diagnosing cancers at earlier stages important, improving patient survival. Our study here provides substantial evidence for establishing ING1 as a probable biomarker for breast cancer. We have shown in this study that ING1 can specifically predict the survival of patients with luminal type breast cancer. Specifically, higher ING1 expression in the stroma leads to poor survival of patients with luminal type breast cancer due to increase in MMP production, which may in turn allow cancer cells to escape and form secondary metastasis. Higher stromal ING1 also establishes a pro-tumor survival niche by virtue of the cytokines and chemokines released by the stromal cells. This phenomenon although anomalous to a typical tumor suppressor, defines a unique role of ING1 in breast cancer pathogenesis. Overall, this study provides important pre-clinical information that may be helpful to evaluate the potential usefulness of the ING family of tumor suppressors in breast cancer prognosis and treatment.

## Material and methods

### Cell culture

Immortalized human mammary fibroblasts HMF3s (a kind gift from Dr. Parmjit Jat) and MCF7 cells were cultured in H-DMEM (Lonza) supplemented with 10 % FBS, 0.1 mg/ml streptomycin and 100 U/ml penicillin and maintained in a humidified atmosphere of 5 % CO_2_ and 95 % air at 37 °C. Cells were routinely tested for mycoplasma contamination. For MMP inhibitor assays, HMF3s cells were treated with 10 μM GM6001 (Millipore) MMP inhibitor alone or in combination with 15 MOI Ad-ING1a or Ad-GFP. Media were collected after 48 h and stored at -80 °C till further use. MCF 7 cells supplemented with conditioned media from these cells were analyzed for changes in morphology after 24 h using a Zeiss Axiovert 200 M microscope.

### Multiplex assay for cytokine and chemokine screening

Media from transfected/infected cells were collected and screened for released cytokines and chemokines using an ELISA based assay (Eve Technologies, Calgary Alberta). Cells (HMF3s) were inoculated overnight in 6 well dishes and were infected with Ad-ING1a or Ad-GFP virus at 15 MOI and allowed to grow for 48 h with media changed 12 h post infection. After 48 h, cell media were carefully collected in sterile centrifuge tubes without disturbing the cells. The media were then centrifuged at 4 °C for 10 min at 13,000 rpm. After centrifugation, the media supernatant were transferred into fresh sterile tubes and stored at −80 °C unless analyzed immediately.

### Three-dimensional cell culture

Three-dimensional culture of HMF3s and MCF7 cells were performed in ultra-low attachment 96-well plates (BD Biosciences). HMF3s cells were infected with Ad-ING1a or Ad-GFP at 15 MOI for 48 h. The plates were firstly coated with 50 μl of 30 % growth factor reduced Matrigel (3 mg/ml) (BD Bioscience) in complete medium (DMEM-containing 10 % FEBS, penicillin, streptomycin and amphotericin B (Lonza) and were incubated for 1 h in a CO_2_ incubator at 37 °C to form a layer. Cell suspensions of HMF3s and MCF7 cells were then made (100 cells/50 μl) in 30 % Matrigel and were carefully layered without formation of bubbles on the solidified layer in the wells. For co-culture experiments, a 1:1 ratio of HMF3s cells and MCF7 cells was used. Fresh media were supplemented every three days and images were taken after 2 weeks using a Zeiss Axiovert 200 M microscope.

### Zymography

To determine the activity of matrix metalloproteases MMP-1 and MMP-2, gelatin and casein zymography were performed, respectively. Briefly, HMF3s cells were grown in 6 well dishes and infected with Ad-ING1a or Ad-GFP for 48 h at 15 MOI and the media was centrifuged at 4 °C for 10 min at 13,000 rpm. The collected media supernatants were then mixed with 2X sample buffer (65.8 mM Tris-HCl pH 6.8, 25 % (w/v) glycerol, 2 % SDS, 0.1 % bromophenol blue) and incubated at room temperature for 10-15 min. The samples were then subjected to zymography by resolving on 10 % SDS- polyacrylamide gels containing 1 mg/ml porcine skin-derived gelatin (Sigma) or casein (Sigma). After electrophoresis, the gels were incubated for 30 min at room temperature with gentle agitation in renaturation buffer (2.5 % Triton X-100) to remove SDS. The gels were then incubated in developing buffer (50 mM Tris-HCl, 0.2 M NaCl, 5 mM CaCl_2_ and 0.02 % Brij 35) at 37 °C overnight with gentle agitation. After incubation in the developing buffer, gels were stained with 0.5 % (w/v) Coomassie Blue R-250 for 30 min and destained with destaining solution methanol : acetic acid : water (50:10:40). For casein zymography, gels were pre-run for 30 min at 100 V before loading samples to remove excess casein.

### Patient cohort and ethics

The Calgary Tamoxifen Cohort which contains a demographic, clinical and pathology data for 819 breast cancer patients diagnosed between 1985 and 2000 at the Tom Baker Cancer Centre in Calgary, Canada was used in this study. Inclusion and exclusion criteria and detailed description of the cohort have been described previously [31]. This study involving the Tamoxifen cohort was ethically approved (Ethics ID E-17422) by the Conjoint Health Research Ethics Board (CHREB) at the University of Calgary, Canada.

### Fluorescence immunohistochemistry and automated image acquisition

Fluorescence immunohistochemistry and image acquisition of the patient samples was performed as described previously [31]. Briefly, 4 μm thick sections were cut from the TMA block and after deparaffinization, were rinsed in ethanol, and rehydrated. Heat-induced epitope retrieval was performed in a decloaking chamber (Biocare Medical, Concord, CA, USA), and slides were stained using a Dako Autostainer. Automated image acquisition was performed using AQUAnalysis® program as described previously [31]. CAb 5 antibody specific to ING1 that can recognize both ING1a and ING1b was used for analyzing ING1 levels in patient samples.

### Assessment of ING1 expression

The average intensity of target ING1 signal in the stromal mask was tabulated and used to generate stromal specific AQUA scores, which reflect the average signal intensity in the stromal area of the tissue sample. The ING1 expression score was defined as the mean ING1 AQUA score from triplicate cores for each patient sample. Patients were dichotomized at the lowest or highest tertiles of ING1 expression within the entire cohort, to define Low ING1 and High ING1 categories.

### Statistical analysis for patient data

Statistical analyses were performed using Stata 12 (StataCorp LP). For survival analysis, disease free survival (DFS) which is defined as the time of diagnosis to recurrence, metastatic disease, or death from breast cancer and disease specific overall survival (DSOS), which is the time of diagnosis to death from breast cancer were analyzed. Patients were censored at the time a patient died from another cause, or the follow-up period ended. Kaplan Meier survival analysis was performed to estimate the probability of 5-year DFS or 7-year DSOS. Cox proportional hazards analyses were conducted to assess the impact of clinical covariates in multivariate analysis.

## Additional file

Additional file 1: Figure S1. Quantitation of 3-D culture colony morphological changes. Colony images from 3-D cultures of HMF3s cells expressing GFP + MCF7 cells and HMF3s cells expressing ING1a + MCF7 cells were visually scored for their levels of disorganization and aggressiveness as estimated by divergence from uniformity (** *p* < 0.001). (DOC 58 kb)
