# Supplementary figures and images for: Stromal ING1 expression induces a secretory phenotype and correlates with breast cancer patient survival
Source: Mol Cancer. 2015 Aug 27;14:164. doi: 10.1186/s12943-015-0434-x (PMC4549945; doi:10.1186/s12943-015-0434-x)

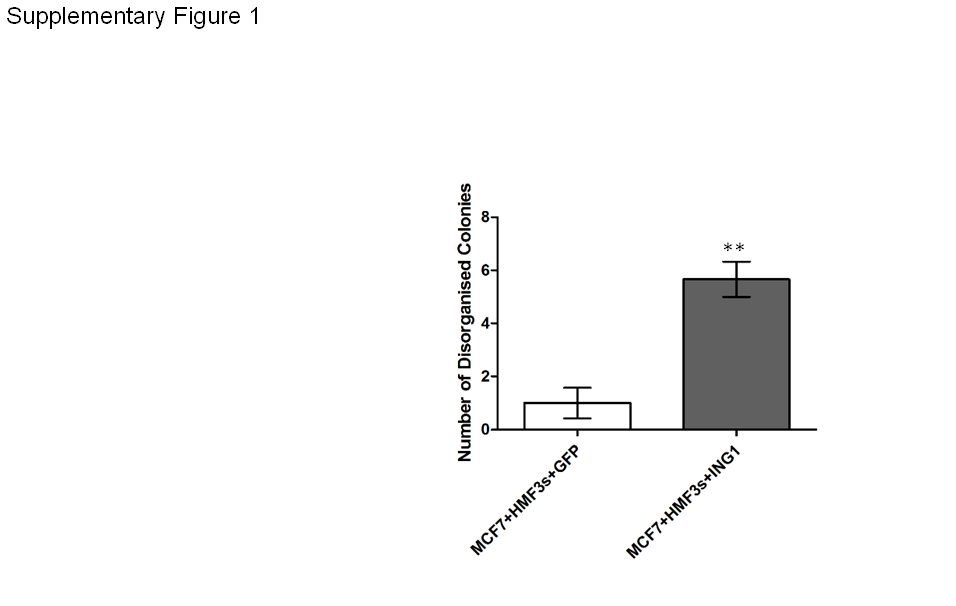

Supplement: Additional file 1: Figure S1. — Quantitation of 3-D culture colony morphological changes. Colony images from 3-D cultures of HMF3s cells expressing GFP + MCF7 cells and HMF3s cells expressing ING1a + MCF7 cells were visually scored for their levels of disorganization and aggressiveness as estimated by divergence from uniformity (** p < 0.001). (DOC 58 kb) [file 12943_2015_434_MOESM1_ESM.doc]
